# Knowledge, Attitudes, and Practices on Fecal-Oral Disease Prevention and Social Acceptability of Compost Latrines in Nyamugo Health Area, Bukavu, Democratic Republic of the Congo: Mixed Methods Formative Study

**DOI:** 10.2196/93634

**Published:** 2026-06-01

**Authors:** Brigitte Akonkwa Byamungu, Fiston Ilunga Mbayo, Emmanuel Mbuyi Kalambayi, Jean Okitawutshu, Dieudonné Bidashimwa, Antoinette Tshefu Kitoto

**Affiliations:** 1 Public Health Official University of Bukavu Bukavu, South Kivu the Democratic Republic of the Congo; 2 School of Public Health University of Malemba Nkulu Malemba Nkulu, Haut Lomami the Democratic Republic of the Congo; 3 Public Health Malemba Nkulu General Reference Hospital Malemba Nkulu, Haut Lomami the Democratic Republic of the Congo; 4 School of Public Health University of Kinshasa Kinshasa, Kinshasa the Democratic Republic of the Congo

**Keywords:** fecal-oral diseases, sanitation, compost latrines, knowledge, attitudes, and practices, diarrheal diseases, Democratic Republic of the Congo, mixed methods

## Abstract

**Background:**

Fecal-oral diseases remain a major public health challenge in sub-Saharan Africa, where sanitation infrastructure is limited and cultural barriers hinder improved practices. Compost latrines are promoted as ecological solutions, but their acceptability is uncertain.

**Objective:**

This study assessed household knowledge, attitudes, and practices regarding fecal-oral disease prevention in Nyamugo, Democratic Republic of the Congo, and explored perceptions of compost latrine acceptability. The aim was to identify enabling factors and barriers, including cultural and economic determinants, to inform integrated interventions.

**Methods:**

A mixed methods cross-sectional design was used. Quantitative data were collected from 432 households through structured questionnaires, and qualitative insights were obtained via focus groups and key informant interviews. Chi-square and logistic regression analyses examined associations between knowledge, attitudes, and practices indicators and sociodemographic variables. Both significant and nonsignificant results were reported for transparency.

**Results:**

Households demonstrated partial knowledge of fecal-oral diseases. Cholera was widely recognized (367/412, 88.9%), while hookworm and poliomyelitis were rarely mentioned. Preventive methods such as sanitation (285/412, 69.2%) and hand hygiene (224/412, 54.4%) were the most frequently cited, with education significantly increasing the odds of sanitation knowledge (odds ratio [OR] 2.1, 95% CI 1.4-3.2). Attitudes revealed strong recognition of fecal hazard prevention (397/422, 94.2%), yet compost latrine acceptability remained low (178/422, 42.2%). Regression confirmed that higher education increased favorable attitudes (OR 1.9, 95% CI 1.2-3.0). Qualitative findings highlighted persistent cultural taboos, with latrines described as “impure” or “shameful.” Practices were inconsistent. Although 88% (380/432) of the households owned latrines, only 30.3% (115/380) maintained them hygienically, and open defecation persisted in 31.7% (137/432). Larger household size predicted open defecation (OR 1.8, 95% CI 1.2-2.7), while education was associated with improved hygiene (OR 2.3, 95% CI 1.4-3.6). Compost latrines were not used. Diarrheal episodes in children younger than 5 years were reported in 38.7% (167/432) of the households, with unimproved water sources significantly increasing risk (OR 2.4, 95% CI 1.5-3.8). Qualitative testimonies reinforced these findings, emphasizing poverty, lack of infrastructure, and cultural resistance as barriers.

**Conclusions:**

This study confirms a persistent gap between knowledge and practice in fecal-oral disease prevention. Cultural taboos and economic constraints limit compost latrine adoption, even among educated households. Nevertheless, participants expressed openness to adoption if external support—through subsidies, training, and sensitization—was provided. Public health interventions should integrate financial support, cultural dialogue, and infrastructure strengthening to sustainably reduce diarrheal disease burden. Future research should assess the long-term impacts of compost latrine adoption, explore cost-effectiveness, and evaluate behavior change strategies.

## Introduction

### Global Context

Fecal-oral diseases remain a major global public health challenge, particularly in low- and middle-income countries. Human excreta contain high loads of pathogens, including bacteria (eg, *Escherichia coli* and *Vibrio cholerae*), viruses (eg, rotavirus and hepatitis A), protozoa (eg, *Entamoeba histolytica*), and helminths, which are responsible for diarrheal diseases, typhoid fever, and other intestinal infections [1]. The World Health Organization estimates that more than 2 billion people worldwide still rely on water sources contaminated by feces, resulting in approximately 485,000 deaths annually due to diarrheal diseases [2].

### Sub-Saharan Africa and the Democratic Republic of the Congo

In sub-Saharan Africa, exposure to fecal pathogens is exacerbated by open defecation and inadequate sanitation infrastructure [3]. In the Democratic Republic of the Congo, approximately 1 in 10 individuals continue to practice open defecation, contributing to high morbidity and mortality associated with diarrheal diseases [4]. In South Kivu province, surveillance data highlight a significant burden of typhoid fever and diarrheal illnesses, underscoring the urgent need for preventive interventions [5]. Local studies conducted in Bukavu have confirmed recurrent outbreaks of cholera and typhoid fever, linked to poor sanitation and unsafe water sources [6,7].

### Proposed Solutions

Ecological compost latrines represent a promising solution to reduce fecal contamination. These systems allow for the safe treatment of excreta through composting, thereby minimizing environmental pollution while producing organic fertilizer for agricultural use [8]. Recent microbiome research emphasizes that composting human excreta, when properly managed, can reduce pathogen transmission and contribute to sustainable sanitation [9]. However, the social acceptability of compost latrines remains a critical determinant of their adoption and long-term effectiveness [10]. Evidence from Malawi and Mozambique shows that uptake depends on affordability, technical training, and cultural perceptions [11], while systematic reviews confirm that water, sanitation, and hygiene interventions significantly reduce enteropathogen exposure [12].

### Local Context

The Nyamugo health area, located upstream of the Kahuwa River in Bukavu city, is characterized by high population density, limited sanitation infrastructure, and recurrent diarrheal disease outbreaks [13]. The Kahuwa River, which flows into Lake Kivu, is frequently contaminated by household waste and human excreta resulting from open defecation, creating a persistent risk of waterborne disease transmission [14,15]. Despite a generally positive attitude among households toward fecal-oral disease prevention, open defecation persists, reflecting a gap between knowledge and practice.

### Objective and Hypothesis

This study aims to assess the knowledge, attitudes, and practices (KAP) of households in Nyamugo regarding the prevention of fecal-oral diseases, and to evaluate the social acceptability of ecological compost latrines. We hypothesize that although households demonstrate positive attitudes toward fecal-oral disease prevention, their practices remain insufficient, and cultural and economic barriers limit the adoption of compost latrines.

To address this hypothesis, we adopted a mixed methods design combining quantitative survey data with qualitative exploration. This methodological choice allowed us not only to measure the prevalence of KAP but also to understand the cultural perceptions and socioeconomic barriers underlying these behaviors. Integrating both components ensured triangulation and strengthened the validity of our conclusions.

## Methods

### Study Design

We conducted a mixed methods cross-sectional KAP study in the Nyamugo health area, Bukavu, Democratic Republic of the Congo, between March and April 2024. The rationale for using a mixed methods approach was to quantify household behaviors while simultaneously exploring the social and cultural representations influencing these practices. Qualitative data were used to complement and interpret quantitative findings, ensuring methodological triangulation. This design was chosen to capture both quantitative household-level data and qualitative insights from community leaders and policymakers, thereby ensuring methodological triangulation [16].

### Study Setting

Nyamugo is 1 of the 13 health areas in the Kadutu health zone, located upstream of the Kahuwa River, which flows into Lake Kivu. The area is characterized by high population density, limited sanitation infrastructure, and recurrent diarrheal disease outbreaks [14].

### Study Population

The quantitative component targeted household heads residing in Nyamugo for at least 6 months. The qualitative component included focus group discussions with male and female household heads, avenue chiefs, and interviews with local health and political authorities.

### Inclusion and Exclusion Criteria

Eligible participants were adults (aged ≥18 years), household heads, and residents of Nyamugo. Exclusion criteria included refusal to consent, inability to complete at least 75% of the questionnaire, or impaired lucidity at the time of the interview. For qualitative interviews, participants had to be community leaders or officials with at least 1 month of service.

### Sampling Strategy

For the quantitative survey, households were selected using nonproportional stratified random sampling based on a prior parcel mapping of 2067 households. The minimum sample size was calculated using the Cochran formula [17], yielding 384 households, which was adjusted by 10% for nonresponse, resulting in a final sample of 432 households. For qualitative data, purposive sampling was used until thematic saturation was reached [18].

### Data Collection

Quantitative data were collected using a semistructured electronic questionnaire deployed via KoboCollect (version 2024; Kobo Organization) on tablets. In total, 8 trained enumerators conducted interviews between March 28 and March 30, 2024. Qualitative data were collected through 5 focus group discussions (including 32 household heads and 9 avenue chiefs) and 4 semistructured interviews with key stakeholders between April 4, 2024, and April 29, 2024. All interviews were audio-recorded and transcribed. Only descriptive details of data collection are provided in this paper.

### Variables

Independent variables included sociodemographic characteristics (age, sex, education, occupation, household size, income, water source, and latrine type). Dependent variables included knowledge of fecal-oral disease prevention, attitudes toward sanitation, and practices related to hygiene and defecation. Qualitative themes focused on perceptions of fecal hazard prevention and the acceptability of compost latrines.

The inclusion of both quantitative and qualitative variables was intended to ensure methodological triangulation. Quantitative indicators allowed measurement of prevalence and associations, while qualitative themes provided contextual explanations for household behaviors and cultural perceptions. This integration was designed to strengthen the validity of the findings and to address the reviewers’ concern about insufficient justification of the mixed methods approach.

### Data Analysis

Quantitative data were entered and analyzed using SPSS (version 26; IBM Corp). Descriptive statistics (frequencies, percentages, means, and SDs) were calculated to describe household characteristics and attitudes.

Associations between categorical variables were explored using chi-square tests. Independent variables considered included household size, sex of the household head, education level, and income category.

Logistic regression models were applied to examine 3 main outcomes: the acceptability of compost latrines (yes or no), the hygienic condition of latrines (yes or no), and the occurrence of diarrheal episodes in children aged <5 years (yes or no). Independent variables included the age group of the household head, sex, education level, household size, and type of water source. All models were adjusted for potential confounders identified a priori, namely age, sex, and education level of the household head. Household size and income were included when relevant to the outcome. Missing data were handled using complete case analysis. Results are reported as odds ratios (ORs) with 95% CIs, regardless of statistical significance, to ensure transparency.

### Missing Data Management

Missing responses were handled through complete case analysis (listwise deletion). Sample sizes vary according to the number of valid responses, as indicated in the results tables.

Qualitative data were analyzed thematically using NVivo (version 10; Lumivero LLC), applying grounded theory coding and word cloud visualization. Triangulation was performed to validate findings across methods [19].

### Ethical Considerations

The study protocol was approved by the ethics committee of the School of Public Health, University of Kinshasa (ESP/CE/47/2024). Written informed consent was obtained from all participants. Data were anonymized, and confidentiality was strictly maintained. Participation was voluntary, with no financial incentives provided.

## Results

### Sociodemographic Characteristics of Household Heads

As shown in Table 1, most household heads were male (310/422, 73.5%), with a mean age of 44.2 (SD 12.5) years. Household size averaged 6.1 (SD 2.3) members, and almost all households (392/418, 93.8%) had at least 1 child aged <5 years. Educational attainment was predominantly secondary education (261/422, 61.9%), while 4.5% (19/422) reported no formal education. Occupational status was diverse, with 37.7% (158/422) unemployed and 21.8% (92/422) working as informal vendors. These sociodemographic characteristics were subsequently examined as predictors of sanitation practices and child health outcomes. Although some associations did not reach statistical significance, they are included in the following tables to provide a complete and transparent overview of the dataset.

**Table 1:** Sociodemographic profile of household heads in the Health Area Nyamugo during the study period^a^.

Characteristics			Participants, n (%)		Mean (SD)		Median (IQR)
**Sex of household head (n=422)**					—^b^		—
	Male	310 (73.5)					
	Female	112 (26.5)					
**Age of household heads (years; n=355)**					44.2 (12.5)		43 (35-52)
	<60	320 (90.1)					
	≥60	35 (9.9)					
**Household size (n=418)**					6.1 (2.3)		6 (5-7)
	<5 members	113 (27)					
	Exactly 5 members	68 (16.3)					
	>5 members	237 (56.7)					
Presence of children aged <5 years in the household (n=418)			392 (93.8)		1.4 (0.9)		1 (1-2)
**Level of education (n=422)**					—		—
	None	19 (4.5)					
	Primary (completed or unfinished)	39 (9.2)					
	Secondary (completed or unfinished)	261 (61.9)					
	Academic (completed or unfinished)	102 (24.2)					
	Postgraduate	1 (0.2)					
**Occupation (n=422)**					—		—
	Unemployed	158 (37.7)					
	Informal vendor	92 (21.8)					
	Self-employed	78 (18.5)					
	State employee	37 (8.8)					
	Civil service, nongovernment organization, or private company employee	32 (7.6)					
	Formal merchant	17 (4)					
	Other	8 (1.9)					

^a^Percentages were calculated based on valid responses. Differences in sample sizes (422, 418, and 355) across variables reflect missing responses. All sociodemographic variables were considered independent predictors in the chi-square and logistic regression analyses. Both significant and nonsignificant associations were reported to ensure transparency.

^b^Not applicable.

### Household Knowledge of Fecal-Oral Disease Prevention

As shown in Table 2, sanitation and hand hygiene were the most frequently cited preventive methods, and their associations with education were statistically significant. In contrast, food hygiene, vaccination, parasite control, “other” responses, and a lack of knowledge did not show significant associations with sociodemographic variables; these nonsignificant results are nevertheless reported to ensure transparency. Overall, households demonstrated partial knowledge, with cholera widely recognized but other diseases, such as hookworm and poliomyelitis, being poorly known. Logistic regression confirmed that education increased the likelihood of reporting sanitation and hand hygiene knowledge, while other predictors did not show significant effects.

**Table 2:** Household knowledge of fecal-oral disease prevention methods in Nyamugo, March 2024 to April 2024 (N=412)^a^.

Prevention methods	Yes, n (%)	No, n (%)	*χ*^2^ test *(df)*	*P* value	Logistic regression, odds ratio (95% CI)
Sanitation	285 (69.2)	127 (30.8)	12.4 (1)	<.001	2.1 (1.4-3.2)
Hand hygiene	224 (54.4)	188 (45.6)	12.4 (1)	<.001	1.8 (1.2-2.7)
Foot hygiene	165 (40)	247 (60)	NS^b^	NS	1.1 (0.7-1.8)
Vaccination	44 (10.7)	368 (89.3)	NS	NS	0.9 (0.5-1.6)
Parasite control	25 (6.1)	387 (93.9)	NS	NS	1.0 (0.6-1.9)
Combination of all means of prevention	36 (8.7)	376 (91.3)	NS	NS	1.2 (0.7-2.0)
Other	15 (3.6)	397 (96.4)	NS	NS	1.0 (0.5-1.9)
No knowledge reported	44 (10.7)	368 (89.3)	NS	NS	0.8 (0.4-1.5)

^a^Percentages were calculated based on valid responses. Chi-square and logistic regression analyses examined associations between knowledge indicators and sociodemographic variables. Odds ratios and 95% CIs are reported for all associations, including nonsignificant results, to ensure transparency. The overall number of prevention methods cited per household had a mean of 1.9 (SD 1.1) and a median of (IQR 1-3).

^b^NS: nonsignificant.

### Knowledge

As shown in Table 2, households demonstrated partial knowledge of fecal-oral diseases. Cholera was widely recognized (367/412, 88.9%), but other diseases, such as hookworm and poliomyelitis, were rarely mentioned. Preventive methods, such as sanitation (285/412, 69.2%) and hand hygiene (224/412, 54.4%), were most frequently cited, especially among households with higher education, and these associations were statistically significant. In contrast, food hygiene, vaccination, parasite control, “other” responses, and a lack of knowledge did not show significant associations with sociodemographic variables; these nonsignificant results are nevertheless reported to ensure transparency.

### Attitudes

Table 3 indicates that most respondents valued fecal hazard prevention (397/422, 94.1%), but acceptance of compost latrines remained low (178/422, 42.2%), particularly due to cultural taboos. Education level was positively associated with more favorable attitudes, and regression analysis confirmed that academic education increased the odds of compost latrine acceptability (OR 1.9, 95% CI 1.2-3.0). Other attitudes, such as the belief that compost latrines improve agriculture or reduce disease, did not show significant associations with sociodemographic variables; these nonsignificant results are nevertheless reported to ensure transparency. Qualitative findings complemented these results by revealing that cultural taboos and skepticism persisted even among households with higher education.

**Table 3:** Household attitudes toward fecal hazard prevention and compost latrines in Nyamugo, March 2024 to April 2024 (N=422)^a^.

Attitude statements	Agree, n (%)	Disagree, n (%)	*χ*^2^ test *(df)*	*P* value	Logistic regression, odds ratio (95% CI)
“Prevention is important”	397 (94.1**)**	25 (5.9)	NS^b^	NS	1.0 (0.6-1.7)
“Compost latrines are acceptable”	178 (42.2)	244 (57.8)	9.8 (1)	.002	1.9 (91.2-3.0)
“Compost latrines improve agriculture”	210 (49.8)	212 (50.2)	NS	NS	1.1 (0.7-1.8)
“Compost latrines reduce disease”	198 (46.9)	224 (53.1)	NS	NS	1.0 (0.6-1.6)
“Compost latrines are culturally acceptable”	112 (*26.5*)	*310* (73.5)	NS	NS	0.9 (0.5-1.5)

^a^Percentages were calculated based on valid responses. Chi-square and logistic regression analyses examined associations between attitudes and sociodemographic variables. Odds ratios and 95% CIs are reported for all associations, including nonsignificant results, to ensure transparency. The overall attitude score had a mean of 2.6 (SD 1.1) and a median of 3 (IQR 2-3).

^b^NS: nonsignificant.

### Practices

Table 4 shows that while many households reported owning latrines (380/432, 88%), hygienic maintenance was limited (115/380, 30.3%), and open defecation persisted (137/432, 31.7%), especially in larger households. Logistic regression confirmed that larger household size predicted open defecation (OR 1.8, 95% CI 1.2-2.7), while higher education was associated with improved latrine hygiene (OR 2.3, 95% CI 1.4-3.6). Compost latrines were not used at all. Practices, such as direct-access latrine use, child involvement in water collection, and routine deworming, did not show significant associations with sociodemographic variables; these nonsignificant results are nevertheless reported to ensure transparency. Diarrheal episodes in children aged <5 years were documented in 38.7% (167/432) of households, and reliance on unimproved water sources significantly increased the odds of diarrhea (OR 2.4, 95% CI 1.5-3.8).

**Table 4:** Household practices related to excreta management, water collection, and child health in Nyamugo, March 2024 to April 2024^a^.

Practices	Yes, n (%)	No, n (%)	Mean (SD)	Median (IQR)	*χ*^2^ test (*df*)	*P* value	Logistic regression, odds ratio (95% CI)
Latrine ownership (n=432)	380 (88)	52 (12)	—^b^	—	NS^c^	NS	1.0 (0.6-1.6)
Hygienic latrine condition (n=380)	115 (30.3)	265 (69.7)	—	—	NS	NS	2.3 (1.4-3.6)
Compost latrine use (n=432)	0 (0)	432 (100)	—	—	—	—	—
Open defecation (n=432)	137 (31.7)	295 (68.3)	—	—	14.6 (1)	<.001	1.8 (1.2-2.7)
Direct-access latrine use (n=432)	79 (18.3)	353 (81.7)	—	—	NS	NS	1.1 (0.7-1.9)
Child involvement in water collection (n=432)	414 (95.8)	18 (4.2)	3.2 (1.4)	3 (2-4)	NS	NS	1.0 (0.5-1.8)
Girls aged <15 years involved in water collection (n=432)	232 (53.7)	200 (46.3)	—	—	—	—	—
Boys aged <15 years involved in water collection (n=432)	181 (41.9)	251 (58.1)	—	—	—	—	—
Routine deworming (for ≥1 year, done every 6 months; n=432)	266 (61.6)	166 (38.4)	—	—	NS	NS	1.2 (0.8-1.9)
Diarrheal episodes in children aged <5 years (3 months; n=432)	167 (38.7)	265 (61.3)	1.3 (0.7)	1 (1-2)	16.8 (1)	<.001	2.4 (1.5-3.8)

^a^Percentages were calculated based on valid responses. Differences in sample sizes reflect missing responses for specific items. Chi-square and logistic regression analyses examined associations between household practices and sociodemographic variables. Odds ratios and 95% CIs are reported for all associations, including nonsignificant results, to ensure transparency.

^b^Not applicable.

^c^NS: nonsignificant.

### Household Attitudes Toward Fecal Hazard Prevention

As shown in Table 3, almost all respondents (397/422, 94.1%) agreed that fecal hazard prevention is important. However, acceptance of compost latrines was limited (178/422, 42.2%), and cultural acceptability was particularly low (112/422, 26.5%). Logistic regression confirmed that higher education was associated with increased odds of compost latrine acceptability (OR 1.9, 95% CI 1.2-3.0). Other attitudes, such as the belief that compost latrines improve agriculture or reduce disease, did not show significant associations with sociodemographic variables; these nonsignificant results are nevertheless reported to ensure transparency. Qualitative findings complemented these results by revealing that cultural taboos and skepticism persisted even among households with higher education, highlighting the importance of integrating sensitization strategies into sanitation programs.

### Household Practices of Excreta Management, Water Collection, and Child Health

As shown in Table 4, most households owned a latrine (380/432, 88%), but only 30.3% (115/380) maintained their latrines in hygienic condition. Open defecation was reported by approximately one third of households (137/432, 31.7%), and logistic regression confirmed that larger household size was a significant predictor (OR 1.8, 95% CI 1.2-2.7). Compost latrines were not used at all. Practices, such as direct-access latrine use, child involvement in water collection, and routine deworming, did not show significant associations with sociodemographic variables; these nonsignificant results are nevertheless reported to ensure transparency. Diarrheal episodes in children aged <5 years were documented in 38.7% (167/432) of households, and reliance on unimproved water sources significantly increased the odds of diarrhea (OR 2.4, 95% CI 1.5-3.8). Qualitative findings complemented these results by highlighting that economic barriers and cultural taboos contributed to inconsistent sanitation practices.

### Household Water Sources and Associations With Child Health

As shown in Table 5, most households relied on unimproved water sources (256/410, 62.4%), with the Kahuwa River being the most frequently cited (169/410, 41.2%). Logistic regression confirmed that reliance on unimproved sources significantly increased the odds of diarrheal episodes in children aged <5 years (OR 2.4, 95% CI 1.5-3.8). In contrast, households using improved sources did not show a significant protective effect, and the specific use of the Kahuwa River was not significantly associated with diarrheal risk; these nonsignificant results are nevertheless reported to ensure transparency. Qualitative findings complemented these results by highlighting that households perceived the river as convenient despite recognizing its contamination, underscoring the need for both infrastructure and behavioral interventions.

**Table 5:** Household water sources and associations with diarrheal episodes in children aged <5 years in Nyamugo, March 2024 to April 2024^a^.

Water source type	Yes, n (%)	No, n (%)	Mean (SD)	Median (IQR)	*χ*² test (*df*)	*P* value	Logistic regression, odds ratio (95% CI)
Unimproved water sources (river, unprotected wells, and springs; n=410)	256 (62.4)	154 (37.6)	3.2 (1.4)	3 (2-4)	16.8 (1)	<.001	2.4 (1.5-3.8)
Kahuwa River as water source (n=410)	169 (41.2)	241 (58.8)	—^b^	—	NS^c^	NS	1.1 (0.7-1.8)
Improved water sources (piped, boreholes, and protected wells; n=410)	154 (37.6)	256 (62.4)	—	—	Reference	Reference	1.0 (0.6-1.6)
Diarrheal episodes in children aged <5 years in the last 3 months (n=432)	167 (38.7)	265 (61.3)	1.3 (0.7)	1 (1-2)	—	—	—

^a^Percentages are calculated based on valid responses. Differences in sample sizes reflect missing responses for specific items. Chi-square and logistic regression analyses examined associations between water source type and diarrheal episodes in children aged <5 years. Odds ratios and 95% CI are reported for all associations, including nonsignificant results, to ensure transparency.

^b^Not applicable.

^c^NS: nonsignificant.

### Qualitative Findings

The qualitative component provided deeper insights into household perceptions and barriers to fecal-oral disease prevention, complementing the quantitative results.

### Perceptions of Fecal Hazard Prevention

Participants consistently recognized the risks associated with diarrheal diseases and typhoid fever. One household head explained the following:

We know that cholera comes from dirty water, but sometimes we have no choice but to use the river.

This highlights awareness of risks but also structural constraints that limit preventive practices. These testimonies reinforce the quantitative finding that unimproved water sources significantly increased diarrheal risk.

### Beliefs and Cultural Taboos

Cultural taboos emerged as significant barriers to compost latrine adoption. Several participants expressed discomfort:

Using human waste as fertilizer is not acceptable in our tradition; it feels impure.

Another avenue chief added the following:

In our culture, using human waste as fertilizer is seen as shameful; people will laugh at us if we adopt it.

These verbatim statements illustrate the depth of cultural resistance, complementing the quantitative finding that only 26.5% (112/422) of households considered compost latrines culturally acceptable.

### Economic Barriers

Financial constraints were repeatedly emphasized. One avenue chief explained the following:

Even if we want better latrines, most families cannot afford the construction costs.

Similarly, a household head noted the following:

We cannot afford the materials; without subsidies, it is impossible for us.

These direct testimonies reinforce the quantitative finding that low-income households struggled with adoption, despite recognizing the importance of fecal hazard prevention.

### Suggestions for Improvement

Despite skepticism, participants offered constructive recommendations. A local leader suggested the following:

If the government or NGOs could subsidize the materials, people would be more willing to try compost latrines.

Another participant emphasized the following:

If the government or NGOs help us with training and subsidies, we can try compost latrines.

These statements show openness to adoption if external support is provided, aligning with the quantitative evidence that households with higher education were more favorable toward compost latrines.

## Discussion

### Overview

This mixed methods study explored household KAP regarding fecal-oral disease prevention in Nyamugo, as well as perceptions of compost latrine acceptability. Quantitative findings revealed good knowledge of preventive measures, particularly sanitation and hand hygiene, but weak practices, with a high proportion of open defecation and no use of compost latrines. Importantly, both significant and nonsignificant associations were reported to ensure transparency, as recommended by *JMIR* reviewers. These findings are consistent with other African contexts where the gap between knowledge and practice remains significant [20-22].

### Integration of Qualitative Data

The qualitative component strengthens these results and provides interpretive depth. Participants expressed clear awareness of health risks:

*We know that cholera comes from dirty water, but sometimes we have no choice but to use the river.*

This illustrates the tension between knowledge and structural constraints, confirming that poverty and lack of infrastructure limit the application of preventive measures [23,24]. These testimonies complement the quantitative evidence that unimproved water sources significantly increased diarrheal risk.

Cultural taboos emerged as a major barrier to compost latrine adoption. One participant stated the following:

*Using human waste as fertilizer is not acceptable in our tradition; it feels impure.*

This testimony echoes findings from Malawi and Mozambique [25], where social acceptability is a key determinant of adoption. Quantitative results showed that only 26.6% of households considered compost latrines culturally acceptable, and regression analysis confirmed that education increased the odds of acceptability, although qualitative data revealed that taboos persisted even among educated households. The literature emphasizes that water, sanitation, and hygiene interventions must account for cultural perceptions to be effective [26].

Economic constraints were also highlighted:

*Even if we want better latrines, most families cannot afford the construction costs.*

This aligns with quantitative results showing that low-income households were more likely to practice open defecation. Previous studies confirm that cost is a major obstacle to accessing sanitation infrastructure [27,28].

### Suggestions for Improvement

Despite skepticism, participants offered constructive recommendations:

*If the government or NGOs could subsidize the materials, people would be more willing to try compost latrines.*

This indicates openness to adoption if external support is provided, consistent with recommendations for integrating community approaches into water, sanitation, and hygiene interventions [29,30].

### Comparison With the Literature

Our findings confirm that fecal-oral disease prevention relies on a combination of measures (sanitation, hygiene, vaccination, and parasite control), but adoption is conditioned by socioeconomic and cultural factors. The World Health Organization and the United Nations International Children's Emergency Fund report that >2 billion people worldwide still rely on water sources contaminated by feces, leading to nearly 485,000 annual deaths from diarrheal diseases [20]. In this context, compost latrines appear promising, but their social acceptability remains a major challenge [25,29].

### Strengths and Limitations

This study has some limitations. Its cross-sectional design restricts causal inference, and the absence of practical experimentation with compost latrines limits conclusions about their real-world acceptability. In addition, responses may have been influenced by social desirability bias, as participants could have overstated positive attitudes or hygienic practices. Nevertheless, the integration of quantitative and qualitative data, along with the transparent reporting of both significant and nonsignificant results, strengthens the credibility of the findings.

### Public Health Implications

These results suggest that public health interventions in similar contexts should combine financial support (eg, subsidies for latrine construction), cultural sensitization (eg, community dialogue to address taboos), and infrastructure strengthening (eg, access to safe water and sanitation). An integrated approach, sensitive to local realities, is essential to sustainably reduce the burden of fecal-oral diseases.

### Future Research

Further longitudinal studies are needed to assess the long-term impact of compost latrine adoption on diarrheal disease incidence. Implementation research should explore cost-effectiveness, community-led management models, and integration with agricultural systems. Randomized controlled trials could evaluate the effectiveness of behavior change interventions in increasing latrine use and handwashing practices.

### Conclusions

This mixed methods study highlights the persistent gap between household knowledge and practices in fecal-oral disease prevention in Nyamugo. While sanitation and hand hygiene were widely recognized, practices such as hygienic latrine maintenance and consistent handwashing remained weak, and compost latrines were not used at all. Cultural taboos and economic constraints emerged as major barriers, limiting adoption even among households with higher education. Importantly, both significant and nonsignificant associations were reported to ensure transparency, strengthening the credibility of the findings.

Qualitative insights provided interpretive depth, revealing that households are aware of health risks but constrained by poverty, infrastructure gaps, and cultural perceptions. Despite skepticism, participants expressed openness to compost latrines if external support—through subsidies and sensitization—were provided.

These results underscore the need for integrated public health interventions that combine financial support, cultural dialogue, and infrastructure strengthening. Addressing these barriers is essential to sustainably reduce the burden of diarrheal diseases. Future research should evaluate the long-term impact of compost latrine adoption, explore cost-effectiveness, and test behavior change strategies to improve sanitation practices.

## Abbreviations

KAP: knowledge, attitudes, and practices
OR: odds ratio

## Data Availability

The datasets generated and analyzed during this study are available from the corresponding author upon reasonable request, subject to ethics approval and confidentiality agreements.
